# Analysis of Seriousness and Preventability of Adverse Drug Reactions in Patients at a Tertiary Care Teaching Hospital: A Case Series

**DOI:** 10.7759/cureus.112392

**Published:** 2026-07-10

**Authors:** Raja Vikram Prasad, Akshay Kumar V, Vivekanandan Kalaiselvan, C Deepalatha, Jaishree Suresh, Sandeep Kodali, Shreya Nallapati, G Jyoshna

**Affiliations:** 1 Department of Community Medicine, Mamata Academy of Medical Sciences, Bachupally, IND; 2 Department of Dermatology, Venerology and Leprosy, Mamata Academy of Medical Sciences, Bachupally, IND; 3 Department of Indian Pharmacopoeia Commission, Ministry of Health and Family Welfare, Government of India, Ghaziabad, IND; 4 Department of Pharmacology, Mamata Academy of Medical Sciences, Bachupally, IND; 5 Department of Emergency Medicine, Mamata Academy of Medical Sciences, Bachupally, IND; 6 Department of Pharmacovigilance Nodal Centre, Mamata Academy of Medical Sciences, Bachupally, IND

**Keywords:** individual case safety reporting, over-the-counter drug, patient safety, pharmacovigilance, severe adverse drug reactions

## Abstract

This case series describes three serious adverse drug reactions (ADRs) reported from tertiary care teaching hospitals and analyses their seriousness and preventability. The cases included fixed drug eruption associated with paracetamol/amoxicillin exposure, Stevens-Johnson syndrome/toxic epidermal necrolysis following ceftriaxone administration, and acute renal failure after intake of a fixed-dose combination of diclofenac and paracetamol. Clinical details, suspected drugs, management, outcomes, and pharmacovigilance reporting were reviewed. Seriousness, causality, and preventability were assessed using standard pharmacovigilance criteria, including the Uppsala Monitoring Centre causality assessment approach. All three cases required hospital-based management, and two involved severe cutaneous adverse reactions requiring multidisciplinary care. The cases highlight the contribution of irrational prescribing, inappropriate drug combinations, and delayed recognition to preventable serious ADRs. Early withdrawal of the suspected drug, timely reporting to pharmacovigilance centres, multidisciplinary management, rational prescribing, adherence to clinical protocols, and patient awareness are essential to reduce recurrence and improve medication safety.

## Introduction

The seriousness of adverse drug reactions (ADRs) includes outcomes such as death, life-threatening reactions, hospitalisation or prolongation of hospitalisation, congenital malformations, and permanent disability [1]. Prolongation of hospitalisation generally refers to an extension of hospital stay by at least 24 hours due to the ADR. Seriousness and severity are related but distinct concepts. Seriousness describes the regulatory or clinical outcome of an ADR, whereas severity describes the clinical intensity of the reaction and may be graded as mild, moderate, or severe using tools such as the Modified Hartwig and Siegel Severity Assessment Scale [2]. Therefore, an ADR may be serious, severe, or both, depending on its outcome and clinical intensity [3].

The incidence of serious ADRs has been reported to range from 6.5% to 20%, while ADRs account for approximately 6.7% of hospitalised patients and 0.32% of fatal hospital outcomes [4]. These epidemiological data are relevant to the present case series because the reported ADRs required hospital-based care, prolonged management, and multidisciplinary intervention. Examples of serious ADRs include liver failure, fatal arrhythmias, anaphylactic shock, Stevens-Johnson syndrome, and toxic epidermal necrolysis, particularly when they are life-threatening, require hospitalisation, prolong hospital stay by at least 24 hours, or result in disability [5,6]. The prevalence of serious ADRs highlights the need to measure disease burden, improve patient safety practices, and plan preventive healthcare strategies [7,8].

The most common drugs associated with severe forms of ADRs include antibiotics such as sulphonamides, penicillins, cephalosporins, and quinolones, as well as anticonvulsants such as phenytoin [9,10]. Severe or serious ADRs can arise from several factors, including irrational medication use, polypharmacy, underlying comorbidities, genetic factors, drug interactions, misuse of over-the-counter drugs, and improper drug combinations [11]. In vulnerable patients, including geriatric patients and those with organ dysfunction or genetic susceptibility, optimising medication use, monitoring, rational healthcare practices, and early ADR recognition may reduce hospital stay, healthcare costs, and long-term disability. Preventing ADRs can also improve patients’ quality of life and treatment outcomes [12,13].

This study presents a case series of three serious ADRs reported from tertiary care teaching hospitals: fixed drug eruption, Stevens-Johnson syndrome/toxic epidermal necrolysis (SJS/TEN), and acute renal failure. To improve case-level clarity, the demographic details, indications for drug administration, suspected drugs, clinical reactions, management, outcomes, seriousness criteria, severity assessment, WHO-Uppsala Monitoring Centre causality categories [14], Modified Schumock and Thornton preventability assessment [15], and pharmacovigilance reporting status of all three cases are presented in a structured tabular format. The objective is to describe the clinical presentation, suspected drugs, management, outcomes, seriousness, severity, causality, and preventability of these cases, and to highlight practical measures to prevent the recurrence of serious ADRs.

## Case presentation

Case 1

A six-year-old male patient presented with fever and sore throat and had received tablet paracetamol and amoxicillin before hospital presentation. Following repeated exposure to these drugs, the patient developed cutaneous eruptions in the form of hyperpigmented patches with central necrosis and erosions involving the face, lips, and trunk. Genitalia, palms, and soles were spared. Based on the temporal association with drug intake, characteristic morphology of well-demarcated hyperpigmented erosive lesions, recurrence after repeated drug exposure, and clinical improvement after withdrawal of the suspected drugs, a diagnosis of fixed drug eruption was made. The suspected drugs were stopped, and the patient was admitted to the intensive care unit for close monitoring. Management included intravenous steroids, fluid therapy, and topical mometasone cream applied twice daily. The case was reported to the Adverse Drug Reaction Monitoring Centre and Pharmacovigilance Centre at Mamata Academy of Medical Sciences (MAMS). The patient was advised to avoid the suspected drugs, including paracetamol and amoxicillin, and any alternative antipyretic or analgesic was advised only after clinician review and risk-benefit assessment. After one month of treatment and follow-up, the patient recovered completely, with healing of all lesions.

Causality assessment was performed using the Uppsala Monitoring Centre causality assessment scale [14] and was considered probable/likely because of the temporal association with drug exposure, improvement after dechallenge, and absence of a more likely alternative explanation. This ADR was classified as serious because the patient required hospital admission and intensive care monitoring. It was considered preventable because repeated exposure to the suspected drug occurred before recognition and withdrawal, and earlier identification with avoidance of re-exposure could have reduced the risk of recurrence. The offending drug was withdrawn, and the patient was managed with intravenous steroids, fluid therapy, and topical mometasone. The total duration from the onset of the cutaneous reaction to complete clinical recovery was approximately one month, after which all lesions had healed. This reaction was classified as a serious and preventable ADR. Following reporting to the Pharmacovigilance Programme of India (PVPI), the case data were entered for further evaluation of possible causes of ADRs and to support necessary regulatory actions, including signal generation. Data retrieved from the VigiLyze database showed that among 1,614 reported cases of similar reactions, 268 were categorised as serious and 911 as non-serious; among the serious outcomes, five were fatal, five were life-threatening, 149 led to prolonged hospitalisation, and one resulted in disability/incapacitation [16]. Individual case safety reports (ICSRs) in this system are submitted in the ICH E2B format. The age range of patients ranged from newborns to individuals aged 75 years. Figure 1 shows an ulcerative skin lesion with raw, eroded tissue and its subsequent post-inflammatory hyperpigmented healing stage.

**Figure 1:**
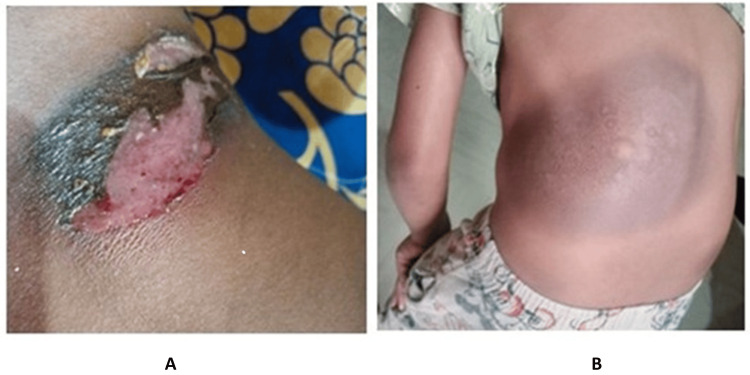
Cutaneous manifestations of fixed drug eruption A. Erosive hyperpigmented plaque with central raw area and post-inflammatory pigmentation. B. Healed lesion showing residual post-inflammatory hyperpigmentation after treatment.

Case 2

A 32-year-old female received an injection of Monocef, containing ceftriaxone 1 g intravenously, for fever, cough, and expectoration. Following ceftriaxone administration, she developed a generalised rash with lesions around the mouth, lips, trunk, and bilateral lower limbs, along with eye congestion. Atypical target lesions were observed over the trunk, and Nikolsky’s sign was positive. The patient was referred to MAMS Hospital, Bachupally, where she was clinically diagnosed with SJS/TEN overlap with multisystem involvement.

The suspected offending drug was promptly withdrawn. A multispecialty approach was initiated, involving Dermatology, General Medicine, Ophthalmology, Gynaecology, ENT, Critical Care, and Pharmacology. Treatment included injection Decadron, containing dexamethasone 4 mg intravenously; injection Pantop, containing pantoprazole 40 mg intravenously; intravenous fluids including DNS and Ringer lactate; Mucaine gel before meals; saline and betadine gargles; mupirocin ointment; Refresh eye drops; tobramycin ophthalmic ointment; Pred Forte eye drops; Lacrigel eye ointment; triamcinolone gel over erosions; liquid paraffin for local application; protein supplementation; calcium 500 mg; prednisolone acetate; azithromycin; and cyclosporine 100 mg. Ryle’s tube feeding was provided because of mucosal involvement and difficulty with oral intake. Since multiple therapeutic interventions were administered simultaneously as part of supportive and multidisciplinary care, the response cannot be attributed to any single treatment modality.

Severity was assessed clinically based on extensive mucocutaneous involvement, ocular involvement, positive Nikolsky’s sign, need for hospital-based multidisciplinary care, and requirement for Ryle’s tube feeding. SCORTEN was not calculated because all required SCORTEN variables were not available in the case record. Using the Modified Hartwig and Siegel Severity Assessment Scale [2], the reaction was considered severe because it required hospitalisation, withdrawal of the suspected drug, intensive multidisciplinary management, and prolonged treatment. Causality assessment was performed using the Uppsala Monitoring Centre causality assessment scale [14] and was considered probable/likely because of the temporal association with drug exposure, improvement after dechallenge, and absence of a more likely alternative explanation. The reaction was considered serious because it required hospitalisation and involved a potentially life-threatening severe cutaneous adverse reaction. The total duration from onset of SJS/TEN manifestations to complete clinical recovery was approximately one month. The patient recovered after one month of treatment. To contextualise the seriousness of similar SJS/TEN-related ADRs, evidence was reviewed from the VigiLyze database. Among 2,403 reported cases of similar SJS/TEN reactions, 1,550 were classified as serious [16]. The clinical manifestations are shown in Figure 2.

**Figure 2:**
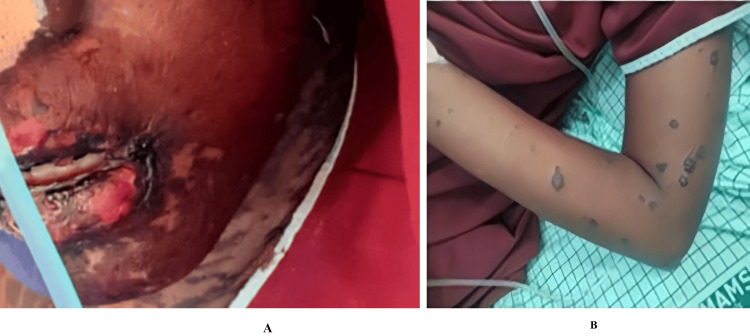
Clinical morphology of Stevens-Johnson syndrome/toxic epidermal necrolysis A. Acute mucocutaneous involvement with erosions and crusting. B. Post-treatment stage showing residual hyperpigmented lesions after clinical recovery.

Case 3

A 30-year-old male patient with back pain received a fixed-dose combination tablet containing diclofenac 50 mg and paracetamol 500 mg. Within 2 hours of taking the medication, he developed swelling of the limbs and generalised body swelling, indicating a clear temporal association between drug exposure and symptom onset. He was admitted to the intensive care unit for further evaluation and management. The patient was diagnosed with acute kidney injury/acute renal failure based on abnormal renal function tests documented in the clinical record. The suspected fixed-dose combination was withdrawn, and supportive treatment was initiated. The patient showed positive dechallenge after withdrawal of the suspected medication and initiation of treatment. Rechallenge was not performed because of the seriousness of the reaction. Management included intravenous fluids and supportive care, furosemide 20 mg intravenously, sodium bicarbonate, and dialysis. Injectable antibiotic therapy was administered as part of inpatient management.

Causality assessment was performed using the Uppsala Monitoring Centre causality assessment scale [14] and was considered probable/likely because of the close temporal association with drug intake, improvement after dechallenge, and lack of a more likely alternative explanation in the available clinical record. Alternative causes of acute kidney injury, including pre-existing renal disease, hypotension, sepsis, dehydration, and exposure to other nephrotoxic drugs, were considered from the available history and clinical documentation; however, detailed laboratory values, urinalysis findings, renal imaging findings, and dialysis indication were not available in the case record. Therefore, the association with diclofenac-paracetamol was interpreted cautiously. This ADR was classified as serious because it required intensive care unit admission and dialysis. It was considered severe based on the requirement for hospital-based intervention and renal replacement therapy. The case highlights the potential risk of acute kidney injury after analgesic exposure, particularly when fixed-dose combinations are used without adequate clinical assessment or monitoring. Awareness among patients regarding appropriate medical consultation and avoidance of unsupervised analgesic use should be strengthened. Table 1 shows the case-wise demographic details, clinical indication, suspected drug, ADR presentation, WHO-UMC causality assessment [14], seriousness classification, Modified Schumock and Thornton preventability assessment [15], and IPC/VigiFlow case-number disclosure status.

**Table 1:** Case-wise summary of adverse drug reactions and pharmacovigilance assessment ADR: adverse drug reaction; IPC: Indian Pharmacopoeia Commission; WHO-UMC: World Health Organization-Uppsala Monitoring Centre. Causality was assessed using the WHO-UMC causality assessment scale [14]. Preventability was assessed using the Modified Schumock and Thornton scale [15]. Unique IPC/VigiFlow case numbers are not included because disclosure of case-level pharmacovigilance identifiers is restricted for confidentiality and patient-safety reporting reasons.

Case	Gender	Age	Clinical condition for drug administration	Suspected drug(s) administered	ADR presentation	WHO-UMC causality assessment	Seriousness	Modified Schumock and Thornton preventability assessment
Case 1	Male	6 years	Fever and sore throat	Amoxicillin 500 mg and paracetamol 500 mg	Fixed drug eruption with skin eruption, hyperpigmentation, central necrosis, and erosions	Probable/likely	Serious	Preventable
Case 2	Female	32 years	Fever, cough, and expectoration	Ceftriaxone 1 g	Stevens-Johnson syndrome/toxic epidermal necrolysis overlap	Probable/likely	Serious	Preventable
Case 3	Male	30 years	Backache	Diclofenac 50 mg and paracetamol 500 mg	Acute renal failure	Probable/likely	Serious	Preventable

## Discussion

Using standard pharmacovigilance assessment, the present cases highlight the dangers associated with misuse, inappropriate prescribing, and delayed recognition of drugs that may result in serious and/or severe ADRs. Because only three cases are presented, the findings should be interpreted as descriptive clinical observations rather than epidemiological estimates or evidence of incidence, prevalence, or population-level risk. In this context, “serious” refers to ADR outcomes such as hospitalisation, intensive care monitoring, prolonged management, disability, or potentially life-threatening reactions, whereas “severe” refers to the clinical intensity of the reaction. Therefore, an ADR may be serious, severe, or both. Fixed drug eruption, acute renal failure, and SJS/TEN illustrate ADRs that may be serious, clinically severe, or both, depending on the patient outcome and clinical presentation. The assessment of seriousness was based on standard pharmacovigilance outcome criteria, while preventability was interpreted using the Modified Schumock and Thornton scale [15] and causality was assessed using the Uppsala Monitoring Centre causality assessment scale [14], according to the available clinical chronology, suspected drug exposure, dechallenge information, and alternative explanations. Table 2 shows the Modified Schumock and Thornton preventability scale [15] categories and corresponding criteria used for ADR preventability assessment.

**Table 2:** Modified Schumock and Thornton preventability scale ADR: adverse drug reaction. Source: [15].

Category	Criteria
Definitely preventable	ADR preventable because a clear avoidable factor was present.
Probably preventable	ADR probably preventable because one or more contributing factors could have been avoided.
Not preventable	ADR not preventable because no identifiable avoidable factor was present.

These cases also emphasise the importance of early identification, prompt withdrawal of suspected drugs, coordinated multidisciplinary management, and cautious prescribing. Patient safety should remain a priority as drug-related complications increase, particularly due to inappropriate drug combinations, misuse of over-the-counter medicines, and polypharmacy. This highlights the need for awareness, rational prescribing, and appropriate clinical management to prevent avoidable adverse outcomes [17]. The present cases are consistent with previously reported concerns that antibiotics, analgesics, fixed-dose combinations, and high-risk prescribing practices may contribute to serious ADRs, especially when early warning signs are missed or when patients receive medications without adequate monitoring. The database, VigiLyze, reported more than 400 million ADR cases worldwide, with nearly 46 million associated with long-term hospitalisation, more than 46 million classified as serious, and more than 300,000 reported as fatal [16,18]. In this manuscript, VigiLyze data were not used for statistical comparison or causal inference; rather, they were included to provide pharmacovigilance context for similar reported ADRs and to demonstrate the broader relevance of individual case safety reporting. The increase in ADRs associated with long hospital stays has been noticeable, with a substantial proportion categorised as serious events [19,20]. Figure 3 presents data retrieved from the Pharmacovigilance Programme of India Performance Report 2023-24 [21], showing the year-wise number of ADR cases leading to prolonged hospitalisation from 2011 to 2024. The figure indicates an overall increasing trend with annual fluctuations, highlighting the need for strengthened ADR reporting, monitoring, and preventive pharmacovigilance measures. 

**Figure 3:**
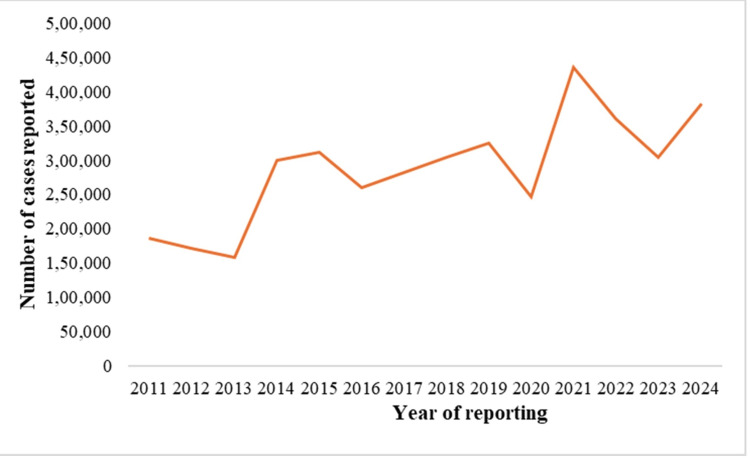
Trend of adverse drug reactions leading to prolonged hospitalisation The figure shows an overall increase in reported cases over time, with fluctuations across individual years and a peak in 2021. These data highlight the continuing burden of serious adverse drug reactions requiring prolonged hospitalisation and support the need for strengthened pharmacovigilance reporting and prevention strategies. Image credit: The authors using Microsoft Excel, based on data from the Pharmacovigilance Programme of India: Performance Report 2023-24, Indian Pharmacopoeia Commission, Ministry of Health and Family Welfare, Government of India [21].

Figure 4 summarises key strategies for preventing and managing ADRs, including rational prescribing, avoidance of unnecessary medications, reduction of polypharmacy, adherence to clinical guidelines, electronic prescribing, structured clinician training, improved ADR reporting, professional accountability, and stronger regulatory oversight.

**Figure 4:**
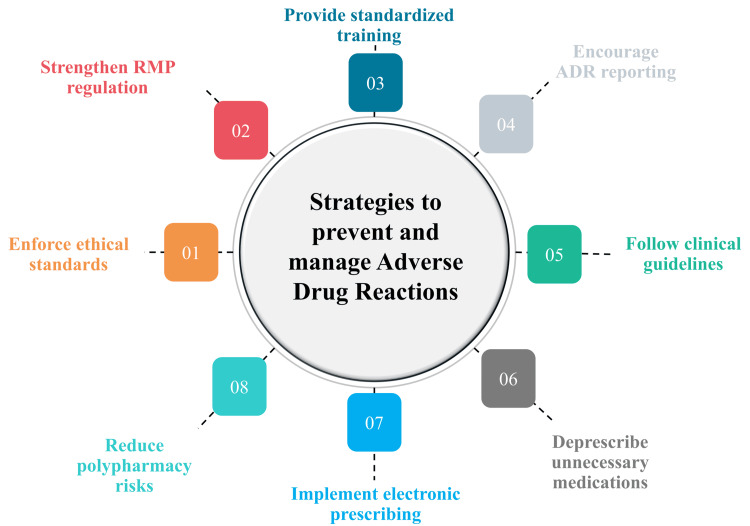
Strategies to prevent and manage adverse drug reactions The figure summarises practical preventive and management strategies relevant to the reported cases, including ethical prescribing, stronger risk-management regulation, structured clinician training, active ADR reporting, adherence to clinical guidelines, deprescribing of unnecessary medications, electronic prescribing, and reduction of polypharmacy-related risk. ADR: adverse drug reaction. Image credit: The authors using Microsoft PowerPoint, based on source [22].

Patient perspective about ADRs

The patient's perspective was documented descriptively from the available clinical history and follow-up information. No validated patient-reported outcome measure, economic burden scale, psychological assessment tool, or quality-of-life instrument was applied; therefore, the findings should be interpreted as descriptive observations rather than quantified patient-reported outcomes. The affected patients experienced treatment-related challenges, including painful mucocutaneous lesions, difficulty with oral intake in cases with mucosal involvement, prolonged hospital-based care, and the need for continued follow-up. These events were associated with physical discomfort, emotional distress for patients and family members, and potential direct and indirect financial burden related to hospitalisation, treatment, travel, and loss of routine activities. Recovery may also be followed by residual pigmentation, anxiety about recurrence, and concern regarding future medication use. These observations highlight the need for patient counselling, clear drug-avoidance advice, early reporting of suspected ADR symptoms, and community awareness regarding safe medication use.

Practitioners' or clinicians' perspective on ADRs 

Clinicians should follow rational therapeutics and evidence-based prescribing practices, with careful assessment of indication, dose, duration, drug interactions, comorbidities, previous drug allergy, and risk-benefit profile before prescribing high-risk medications or fixed-dose combinations. In tertiary care settings, suspected ADRs should be documented in the case record, the suspected drug should be withdrawn when clinically appropriate, and serious reactions should be reported promptly to the Pharmacovigilance Centre through ICSRs [20]. Clinical teams should also provide written drug-avoidance advice to patients, document suspected culprit drugs clearly at discharge, and communicate ADR history during referrals and follow-up visits.

Implementation can be strengthened through routine prescription audits, electronic alerts for drug allergies and high-risk combinations, periodic clinician training, multidisciplinary review of serious ADRs, and integration of ADR reporting into ward rounds and discharge workflows. Common barriers to ADR reporting in tertiary care hospitals include lack of time, uncertainty about causality, incomplete documentation, limited awareness of reporting procedures, fear of blame, and under-recognition of delayed or atypical reactions. Addressing these barriers through simplified reporting systems, feedback from pharmacovigilance centres, and a non-punitive reporting culture may improve ADR detection, reporting quality, and patient safety.

Regulators' perspective on ADRs

From a regulatory perspective, the reported cases demonstrate the importance of complete ADR documentation, timely case submission, case validation, product and event coding, causality assessment, seriousness classification, and quality review. In the present cases, accurate identification of the suspected drugs, clinical chronology, dechallenge information, seriousness criteria, and patient outcomes is essential for generating reliable ICSRs. Such case-level information allows pharmacovigilance centres and regulatory authorities to identify preventable patterns, including repeated exposure to suspected drugs, inappropriate analgesic or antibiotic use, fixed-dose combination-related risk, and delayed recognition of serious cutaneous or renal reactions. These regulatory processes directly support patient safety by enabling signal detection, follow-up queries, risk communication, and timely regulatory advisories when recurring patterns are observed. Strengthening the quality of case narratives, ensuring complete clinical details, and improving follow-up documentation can enhance the scientific value of reports submitted to pharmacovigilance databases and improve the prevention of serious ADRs.

Challenges and recommendations

The major challenges identified from these cases include delayed recognition of ADRs, incomplete documentation of drug exposure and dechallenge, under-reporting to pharmacovigilance centres, use of potentially inappropriate drug combinations, and limited patient awareness regarding early warning symptoms. In routine tertiary-care practice, interventions should be prioritised according to feasibility and clinical impact. High-priority and feasible interventions include systematic documentation of suspected ADRs in case records, prompt withdrawal of suspected drugs when clinically appropriate, timely reporting to the pharmacovigilance centre, clear discharge counselling regarding drugs to avoid, and written documentation of suspected culprit drugs. Regular prescription audits, clinician training, medication reconciliation, and multidisciplinary review of serious ADRs should be incorporated into hospital practice to reduce preventable harm. Moderate-resource interventions include electronic prescribing systems, electronic alerts for documented drug allergies or high-risk drug combinations, and structured ADR reporting workflows integrated into ward rounds and discharge processes. Pharmacogenomic testing and therapeutic drug monitoring may be useful for selected high-risk drugs or patients, but their routine implementation in all tertiary-care settings may be limited by cost, infrastructure, laboratory availability, and trained personnel. Therefore, these strategies should be applied selectively where clinically indicated and practically feasible. Community awareness programmes should also emphasise avoidance of unsupervised medication use and early presentation to registered healthcare facilities when suspected ADR symptoms occur.

## Conclusions

This case series describes three serious ADRs presenting as fixed drug eruption, SJS/TEN, and acute renal failure. Observations derived directly from these cases include temporal association with suspected drug exposure, need for hospital-based care, intensive monitoring, multidisciplinary management or dialysis, and clinical improvement after withdrawal of the suspected drugs with supportive treatment. These findings highlight the importance of early recognition, timely dechallenge, structured causality assessment, and careful documentation of suspected ADRs. Beyond the three reported cases, general pharmacovigilance principles support prompt ADR reporting, rational prescribing, avoidance of unnecessary fixed-dose combinations and polypharmacy, prescription review, and patient counselling regarding suspected culprit drugs. These broader measures may help reduce preventable ADR-related harm but should be interpreted as general recommendations rather than conclusions proven by this small descriptive case series. The findings should therefore be interpreted cautiously in view of the limited number of cases and the descriptive nature of the report.

## References

[1] Adhikari P, Singh U, Yadav PS, Shah L, Dev A, Mandal SC (2024). A young female's battle with toxic epidermal necrolysis induced by NSAIDs: A case report. Ann Med Surg (Lond).

[2] Hartwig SC, Siegel J, Schneider PJ (1992). Preventability and severity assessment in reporting adverse drug reactions. Am J Hosp Pharm.

[3] Patton K, Borshoff DC (2018). Adverse drug reactions. Anaesthesia.

[4] Edwards IR, Aronson JK (2000). Adverse drug reactions: Definitions, diagnosis, and management. Lancet.

[5] Teo YX, Walsh SA (2016). Severe adverse drug reactions. Clin Med (Lond).

[6] Yang S, Kar S (2023). Application of artificial intelligence and machine learning in early detection of adverse drug reactions (ADRs) and drug-induced toxicity. Artif Intell Chem.

[7] Cacabelos R, Naidoo V, Corzo L, Cacabelos N, Carril JC (2021). Genophenotypic factors and pharmacogenomics in adverse drug reactions. Int J Mol Sci.

[8] Kalaiselvan V, Thota P, Singh GN (2016). Pharmacovigilance Programme of India: Recent developments and future perspectives. Indian J Pharmacol.

[9] Wolf M Jr, Rolf J, Nelson D, Smith D, Hess E (2023). Evaluation of detected medication errors within the operating room at an academic medical center. Hosp Pharm.

[10] Grover IS, Deepak C, Prasad RK, Satyagama S (2021). Analysis of drug use in patients of analgesic nephropathy. Natl J Physiol Pharm Pharmacol.

[11] Ciddhavaduta DL, Kalaiselvan V, Midde ML, Baloju D (2020). Analysis of severity and preventability in patients of-Toxic epidermal necrolysis-A case series. Clin Case Rep.

[12] Khalil H, Huang C (2020). Adverse drug reactions in primary care: A scoping review. BMC Health Serv Res.

[13] Pirmohamed M, James S, Meakin S (2004). Adverse drug reactions as cause of admission to hospital: Prospective analysis of 18 820 patients. BMJ.

[14] Uppsala Monitoring Centre (2026). The Use of the WHO-UMC System for Standardised Case Causality Assessment. Uppsala Monitoring Centre. https://who-umc.org/media/u4gjgxvv/who-umc-causality-assessment_new-logo.pdf.

[15] Schumock GT, Thornton JP (1992). Focusing on the preventability of adverse drug reactions. Hosp Pharm.

[16] (2026). VigiLyze Data Mining Tool. Uppsala Monitoring Centre. https://vigilyze.who-umc.org/.

[17] Wolfe D, Yazdi F, Kanji S (2018). Incidence, causes, and consequences of preventable adverse drug reactions occurring in inpatients: A systematic review of systematic reviews. PLoS One.

[18] Insani WN, Whittlesea C, Alwafi H, Man KK, Chapman S, Wei L (2021). Prevalence of adverse drug reactions in the primary care setting: A systematic review and meta-analysis. PLoS One.

[19] Agbabiaka TB, Savović J, Ernst E (2008). Methods for causality assessment of adverse drug reactions: A systematic review. Drug Saf.

[20] Singh P, Verma S, Vaishnav Y, Joshi U, Agrawal M (2025). Probing the in-depth analysis of serious adverse drug reactions in a tertiary care hospital of Central India. Explor Res Clin Soc Pharm.

[21] (2025). Pharmacovigilance Programme of India: Performance Report 2023-24. Indian Pharmacopoeia Commission, Ministry of Health and Family Welfare, Government of India. https://www.ipc.gov.in/images/Annual_Performance_Report_of_PvPI_2023-2024.pdf.

[22] Aate J (2024). Management of adverse drug reactions: A review. IP Int J Compr Adv Pharmacol.

